# Dupuytren’s contracture in a young male: a rare clinical image

**DOI:** 10.11604/pamj.2022.41.341.34745

**Published:** 2022-04-27

**Authors:** Mayur Bhaskar Wanjari, Tejaswee Lohakare

**Affiliations:** 1 Department of Research and Development, Jawaharlal Nehru Medical College, Datta Meghe Institute of Medical Sciences, Sawangi, Wardha, Maharashtra, India,; 2 Department of Child Health Nursing, Smt. Radhikabai Meghe Memorial College of Nursing, Datta Meghe Institute of Medical Sciences, Sawangi, Wardha, Maharashtra, India

**Keywords:** Dupuytren’s disease, fibroproliferative, genetic

## Image in medicine

Dupuytren's disease is a benign fibroproliferative disorder involving the palmar and digital fascia; manifestation depends on genetic and environmental factors. We are presenting a case of a 32-year-old male; he comes to the emergency department with a complaint of limited extension of the little, ring and middle finger from one month, and there are no family and genetic history regarding Dupuytren's disease. More recently has been linked with occupational work, which has been attributed to exposure of hand transmitted vibration. On physical examination patient, both hands little, ring and middle finger are stretched; finding on the examination revealed Dupuytren's disease.

**Figure 1:**
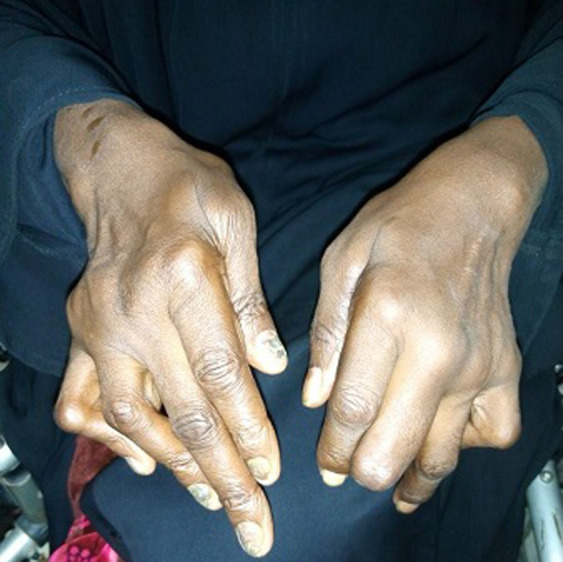
both hand contractures in the little, ring and middle finger

